# Real-Time and High-Resolution 3D Face Measurement via a Smart Active Optical Sensor

**DOI:** 10.3390/s17040734

**Published:** 2017-03-31

**Authors:** Yong You, Yang Shen, Guocai Zhang, Xiuwen Xing

**Affiliations:** School of Applied Science and Civil Engineering, Beijing Institute of Technology, Zhuhai 519088, China; 12057@zhbit.com (Y.Y.); 12031@zhbit.com (G.Z.); 12009@zhbit.com (X.X.)

**Keywords:** active optical sensor, structured light, composite pattern, 3D face measurement

## Abstract

The 3D measuring range and accuracy in traditional active optical sensing, such as Fourier transform profilometry, are influenced by the zero frequency of the captured patterns. The phase-shifting technique is commonly applied to remove the zero component. However, this phase-shifting method must capture several fringe patterns with phase difference, thereby influencing the real-time performance. This study introduces a smart active optical sensor, in which a composite pattern is utilized. The composite pattern efficiently combines several phase-shifting fringes and carrier frequencies. The method can remove zero frequency by using only one pattern. Model face reconstruction and human face measurement were employed to study the validity and feasibility of this method. Results show no distinct decrease in the precision of the novel method unlike the traditional phase-shifting method. The texture mapping technique was utilized to reconstruct a nature-appearance 3D digital face.

## 1. Introduction

The accurate acquisition of 3D face appearance characteristics is important to plan facial contouring surgery, and excellent work is based on an exact 3D face modeling [1,2]. Experts want to devise a non-contact, rapid, and precise approach to acquiring a 3D digital face profile, which is applied to simulate and design an optimal plan for face surgery by modern technologies, such as computer-aided design [3,4,5].

Three types of 3D face modeling methods are currently employed to extract human face profiles: computer tomography (CT) technology [4,5,6,7,8,9,10], the passive optical 3D sensing technique [11,12,13,14,15,16,17,18,19,20,21,22,23], and the active optical 3D sensing technique [24,25,26,27,28,29,30,31,32,33]. The 3D reconstruction method based on CT technology is sensitive to the skeleton and can be conveniently utilized for craniofacial plastics, as well as the oral and maxillofacial correction of abnormalities [4,5,6,7,8,9,10]. However, CT technology cannot easily rebuild soft tissue 3D profiles and obtain human face surface features.

Passive optical 3D sensing techniques, such as stereo vision, utilize two or more camera systems to capture a scene in ambient light from different viewpoints. They determine the height by matching the image features of the corresponding surface features. This method requires many factors to be noticed, such as ambient light, background, vision angle, face gesture, expression, and shading, because they can directly influence measuring accuracy. Furthermore, a mass of data operations, such as correlation analysis and matching operations, are also necessary. Along with the development of computing techniques, the arithmetic speed is no longer a key limiting factor, and the passive optical 3D sensing technique is more often applied for 3D object recognition and understanding. Recently, there have been promising passive long-wavelength infrared polarimetric imaging techniques for 3D face reconstruction; for example, American scholars Alex J. Yuffa and his team proposed an interesting approach whereby a 3D image of a human face is obtained from Stokes parameters and the degree of linear polarization of long-wavelength infrared radiation emitted by human faces [23].

The active optical 3D sensing technique employs structured light to illuminate the specimen. The time or space in the structured light is modulated by the height, and the 3D information can then be extracted from the observation light by a certain unwrapping algorithm [24,25]. Given its non-contact, high-resolution, and highly automated features, the active optical 3D sensing technique is employed in most 3D sensing systems for 3D surface-shape measurement [24,25,26,27,28,29,30,31,32,33].

Phase measuring profilometry (PMP) is an important method of the active optical 3D sensing technique [26,27]. The sinusoidal fringe and phase-shifting techniques are employed in PMP to obtain the desired height information. A flaw in PMP is that it has to capture at least three continuously modulated phase-shifting fringes that correspond to a static profile. Therefore, real-time dynamic measurement becomes difficult, and slight movements or facial expression changes of the target human face potentially bring errors to the demodulated results during the shooting process. A series of phase-shifting fringes can be projected and shot within a short span of time by utilizing a fast digital grating projection approach. However, the images photographed by a charge-coupled device (CCD) camera can easily cause drawbacks, such as trailing and distortion, due to the rapid rotation of the phase-shifting fringe. The measurement inaccuracy also increases. Thus, a one-shot technique is now becoming the trend [31,32,33].

This paper reports on a novel one-shot approach for 3D human face profile measurement. A composite pattern (CP) is adopted in place of the series phase-shifting fringe in PMP, and only a single CP frame is required for projection and capture. The CP efficiently combines several phase-shifting fringes and the same number of carrier gratings so that the phase-shifting technique can also be utilized in this approach. The method to generate this type of composite pattern is presented in Section 2, and the measuring principle and demodulation approaches are also provided in this section. Section 3 presents experimental tests to prove the validity and feasibility of the proposed method. Difficulties and outlooks are then discussed in Section 4.

## 2. Measuring System Based on a Composite Pattern

### 2.1. Composite Pattern of the Phase-Shifting Fringes and Carrier Gratings

**Figure 1:**
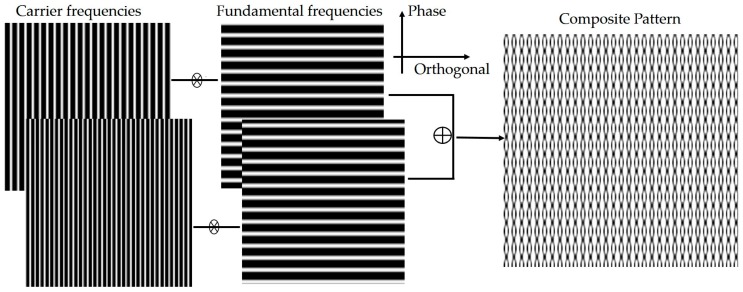
Composite pattern (CP) composed by the phase-shifting fringes and carrier gratings.

A CP is composed of two phase-shifting sinusoidal fringes with the same frequency (called fundamental frequency) and two carrier gratings with distinct frequencies (called carrier frequencies). Each of the two phase-shifting fringes, which are along the depth distortion direction (i.e., phase direction), is modulated by a carrier fringe along the orthogonal axis of the phase direction (i.e., orthogonal direction). They are then combined to obtain the CP, as shown in Figure 1.

The phase-shifting sinusoidal fringe in CP is given as follows:

(1) $$G_{n}\;=\;c+\cos(2\;\pi \;f_{\phi }\;y\;+\;\pi \;n)$$

where constant c is applied to offset $G_{n}$ to be non-negative values, $f_{\phi }$ is the fundamental frequency, y is the phase direction, and n is the phase-shifting index from 0 to 1. The above figure describes CP as follows:

(2) $$I(x,y)=a+b\{[c+\cos(2\pi f_{\phi }y)]\cos(2\pi f_{1}x)+[c+\cos(2\pi f_{\phi }y+\pi )]\cos(2\pi f_{2}\;x)\}$$

where $f_{1}$ and $f_{2}$ are the carrier frequencies of the carrier gratings along the orthogonal direction, and a and b are the projection constants.

### 2.2. Projection and Modulation

The proper values of the projection constants are selected to ensure that the projection intensity of CP falls within I(x,y). A CP is projected by a digital light processor (DLP) to cover the target human face so that the surface can be captured as an entire field recording. Given that CP phases are modulated by height, the intensity of the reflected light field is changed as follows:

(3) $$P(x,y)=ar(x,y)+br(x,y)\{[c+\cos(2\pi f_{\phi }\;y+\varphi (x,y))]\cos(2\pi f_{1}\;x)+[c+\cos(2\pi f_{\phi }\;y+\varphi (x,y)+\pi )]\cos(2\pi f_{2}\;x)\}$$

where r(x,y) is the reflectivity, and φ(x,y) is the wrapped phase related to the face depth. This modulated distortion pattern is captured by a CCD camera.

### 2.3. Phase Acquisition and Unwrapping

After 2D Fourier transform (FT) and simplification, Equation (3) can be rewritten as follows:

(4) $$\begin{array}{l} F(\xi ,\eta )\;=\;A(\xi ,\eta )\;+\;\frac{1}{2}\{\;\\ {}[B(\xi -f_{1},\eta )+\psi (\xi -f_{1},\eta -f_{\phi })+\psi^{*}(\xi -f_{1},\eta +f_{\phi })]+\\ {}[B(\xi +f_{1},\eta )+\psi (\xi +f_{1},\eta -f_{\phi })+\psi^{*}(\xi +f_{1},\eta +f_{\phi })]+\\ {}[B(\xi -f_{2},\eta )-\psi (\xi -f_{2},\eta -f_{\phi })-\psi^{*}(\xi -f_{2},\eta +f_{\phi })]+\\ \left[B(\xi +f_{2},\eta )-\psi (\xi +f_{2},\eta -f_{\phi })-\psi^{*}(\xi +f_{2},\eta +f_{\phi })]\;\right\} \end{array}$$

where F(ξ,η), A(ξ,η), B(ξ,η), and ψ(ξ,η) represent the 2D Fourier frequency spectra of P(x,y), ar(x,y), bcr(x,y), and $\frac{1}{2}br(x,y)\exp[j\varphi (x,y)]$, respectively, and ξ and η correspond to the orthogonal and phase directions in the Fourier domain. The symbol * in the equation represents ψ* is the conjugate function of ψ. The spectrum A(ξ,η) is located in the center of the frequency domain, which is called zero frequency spectra.

Equation (4) indicates that the two carrier frequency spectra and their conjugates are evenly distributed in both sides of the zero frequency spectra. A suitable 2D band-pass filter (e.g., Hanning window) was employed to separate each channel, and then inverse FT (IFT) was performed on each channel. Their absolute values were obtained, and the demodulated phase-shifting fringe was then derived. Grayscale adjustment was then introduced to the fringe, such that

(5) $$\left\{ \begin{array}{l} g_{1}(x,y)=ar^{\prime }(x,y)+br^{\prime }(x,y)\times \cos[2\pi f_{\phi }y+\varphi (x,y)]\\ g_{2}(x,y)=ar^{\prime }(x,y)+br^{\prime }(x,y)\times \cos[2\pi f_{\phi }y+\varphi (x,y)+\pi ] \end{array}\right.$$

where $r^{\prime }(x,y)$ is a constant matrix that represents the reflectivity. Subtraction between the phase-shifting fringes is performed, such that

(6) $$g(x,y)=2br^{\prime }(x,y)\cos[2\pi f_{\phi }y+\varphi (x,y)].$$

**Figure 2:**
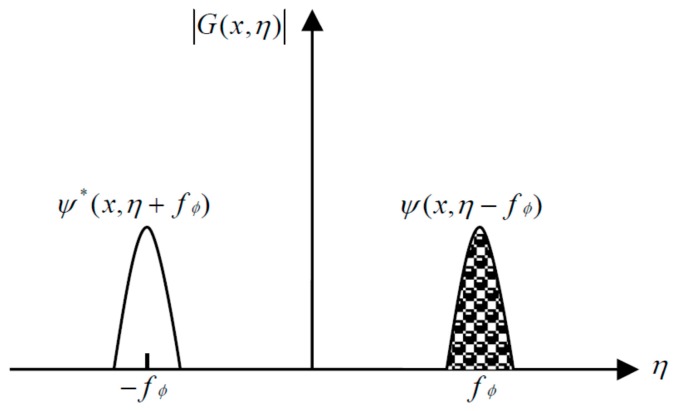
Spectral distribution.

After 1D FT is conducted along the y-direction (viz. phase direction), Equation (6) can be rewritten as follows:

(7) $$G(x,\eta )=\psi (x,\eta -f_{\phi })+\psi^{*}(x,\eta +f_{\phi })$$

where G(x,η) and ψ(x,η) represent the 1D Fourier frequency spectra of g(x,y) and $br^{\prime }(x,y)\exp[i\varphi (x,y)]$, respectively. Figure 2 shows the distribution of the frequency spectra G(x,η).

A proper band-pass filter is selected to extract the fundamental component, which is shown in Figure 2 as a shadow. The wrapped phase φ(x,y) related to the face depth can then be obtained from the fundamental component by performing IFT. A reference plane is required behind the target face to determine the true depth. By utilizing the aforementioned method, the wrapped phase $\varphi_{0}(x,y)$ related to the reference plane can be obtained beforehand. The wrapped phase values of φ(x,y) and $\varphi_{0}(x,y)$ require an unwrapping process to connect the interrupted phases caused by an inversely tangential or imaginary phase algorithm. The height values h(x,y) of the human face can finally be calculated as follows [24,25,26,27]:

(8) $$h(x,y)=\frac{1}{2\pi f_{\phi }}(\frac{L_{0}}{d})\Delta \varphi (x,y)=\frac{1}{2\pi f_{\phi }}(\frac{L_{0}}{d})[\varphi (x,y)-\varphi_{0}(x,y)]$$

where $\frac{L_{0}}{d}$ represents the angle parameters between the CCD camera and DLP (viz. the geometric parameters of the system).

### 2.4. Working Process and Optical Geometric Parameters

**Figure 3:**
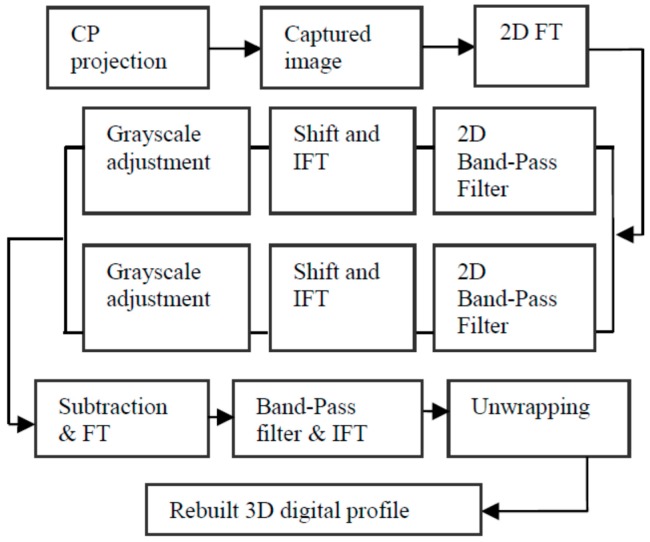
Block diagram of the system process.

Given the aforementioned measuring principle, the working process can be described as follows: (1) several phase-shifting fringes and carrier gratings are combined to generate a CP, which is then projected to cover the target human face by DLP; (2) the CP phases (viz. carrier signals) are modulated by the face depth (viz. modulation signals), and the modulated distortion pattern is then captured by a CCD camera (imaging system); (3) the captured picture is sent to a computer for processing; (4) a series of calculations and a certain unwrapping algorithm obtains the unwrapped phases; and (5) the target 3D human face profile is obtained according to the relationship between phase and height. Figure 3 shows the process details.

**Figure 4:**
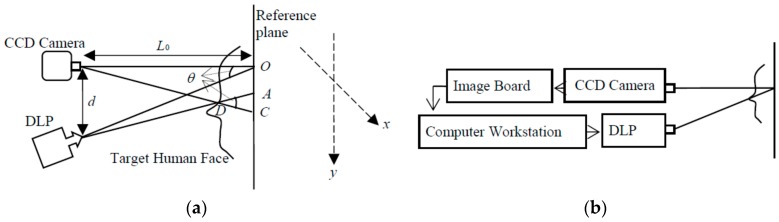
(**a**) Optical geometry of the system and (**b**) a diagram of the experimental setup.

The optical geometric parameters are shown in Figure 4a, where d is the instance between CCD and DLP, and $L_{0}$ is the distance from CCD to the reference plane. Figure 4b shows the diagram of the experimental setup.

## 3. Experiment and Results

### 3.1. Model Test

**Figure 5:**
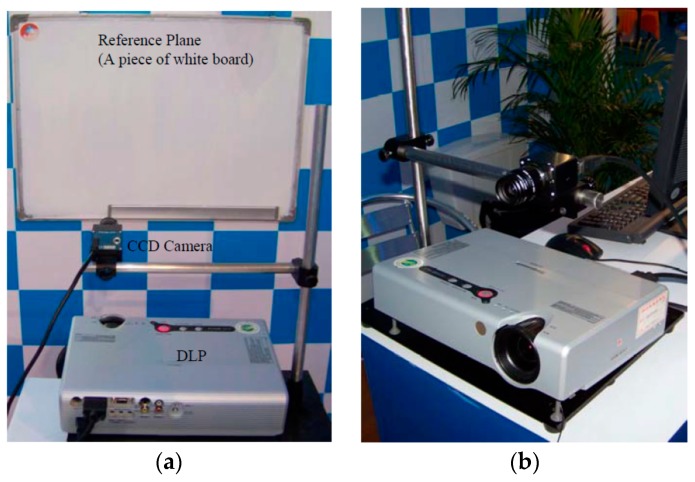
Experimental setup. (**a**) A general view of the setup; (**b**) the digital light processor (DLP) and the CCD camera used in the experiments.

A human face model was utilized as a tentative test to demonstrate the feasibility of this one-shot technique. The projector used was a Panasonic (PT-P2500) digital projector with a 1024 × 768 resolution. The image sensor utilized was a low-aberrance color CCD camera (Prosilica, EC1350C, Vancouver, BC, Canada) with a 1360 × 1024 resolution, pixel size of 4.65 μm × 1.65 μm, and maximum frame rate of 18 fps. The focus of the camera lens (KOWA, LM12JCM, Nagoya, Japan) was 12 mm. The image board was a 1394 card (KEC, 1582T, Taiwan). The reference plane was a piece of white board. Figure 5 shows the experimental setup, where the geometric parameters were detected as $L_{0}$ = 73 mm and d = 18 mm.

**Figure 6:**
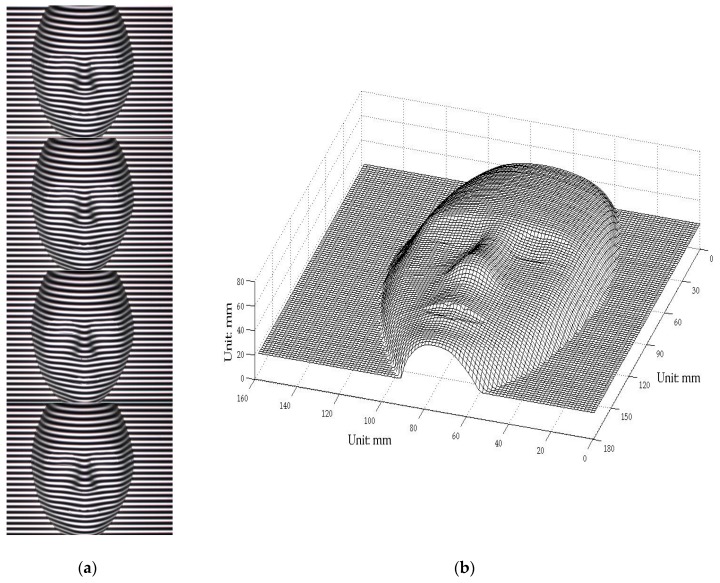
(**a**) Captured four fringes with a phase-shifting value of π/4 modulated by the model surface; (**b**) rebuilt 3D model profile based on phase measuring profilometry (PMP).

The traditional PMP method (i.e., the four-step phase-shifting technique) was first adopted to rebuild the 3D face model. The fundamental frequency $f_{0}$ in PMP was 1/25 line/pixel. Figure 6a shows the captured four phase-shifting fringes, which were modulated by the face model surface depth. Figure 6b shows the rebuilt results utilizing PMP. A suitable rebuilt 3D shape could be obtained utilizing a static face model.

**Figure 7:**
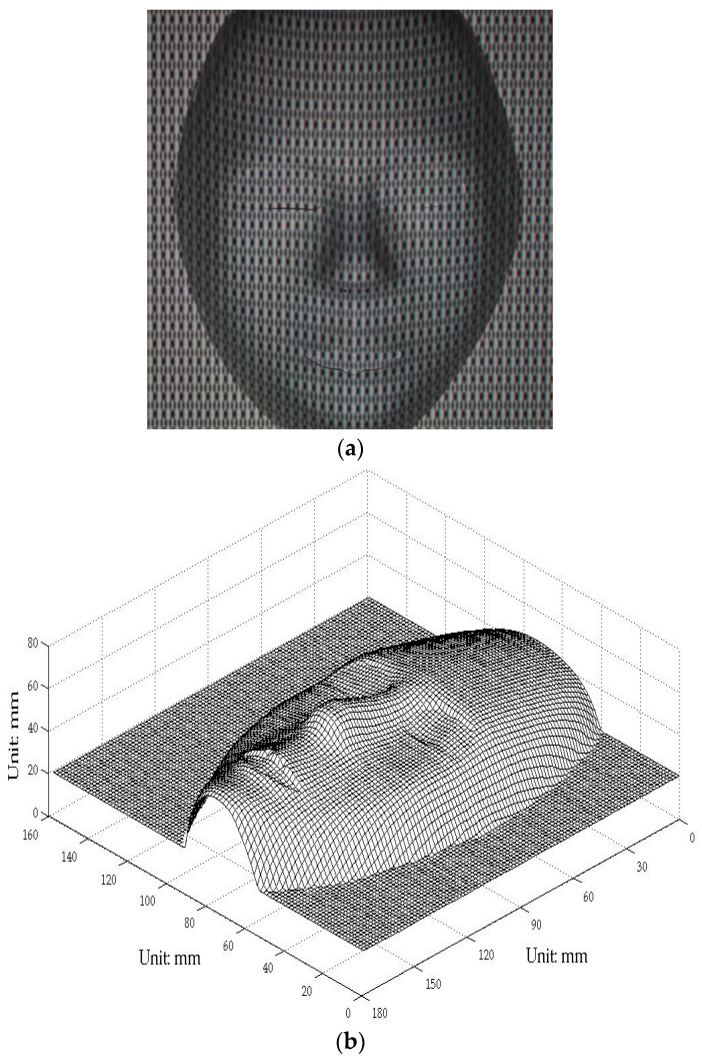
(**a**) Captured distortion composite pattern modulated by the model; (**b**) rebuilt 3D model profile from the composite pattern.

**Figure 8:**
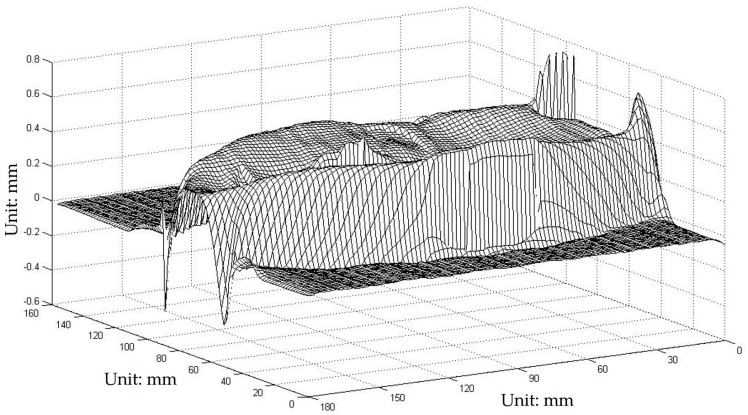
Errors of the proposed one-shot 3D reconstruction against traditional method.

The composite pattern was then introduced as a one-shot technique to reconstruct the face model and compare the results. The carrier frequencies $f_{1}$ and $f_{2}$ were 1/10 line/pixel and 1/5 line/pixel, respectively, whereas the fundamental frequency $f_{\phi }$ was 1/20 line/pixel. The composite pattern coding, projection, and data acquisition, storage, and processing were controlled utilizing a computer workstation. Figure 7a shows the captured distortion composite pattern modulated by height. Figure 7b shows the obtained 3D digital profile of the face model. The images show no noise point, and the reconstructed surface clearly shows the entire face profile with a suitable resolution. Notably, this approach is faster and more convenient because it requires only one capture. Figure 8 shows the errors of the proposed one-shot 3D reconstruction against the traditional PMP method. The small range of the errors from −0.6428 mm to 0.7823 mm show that they were in reasonable agreement. We found that the error is bigger around the edge of the face, which shows that the algorithm has a limit around the area with discontinuities in surface slopes due to sharp edges.

**Figure 9:**
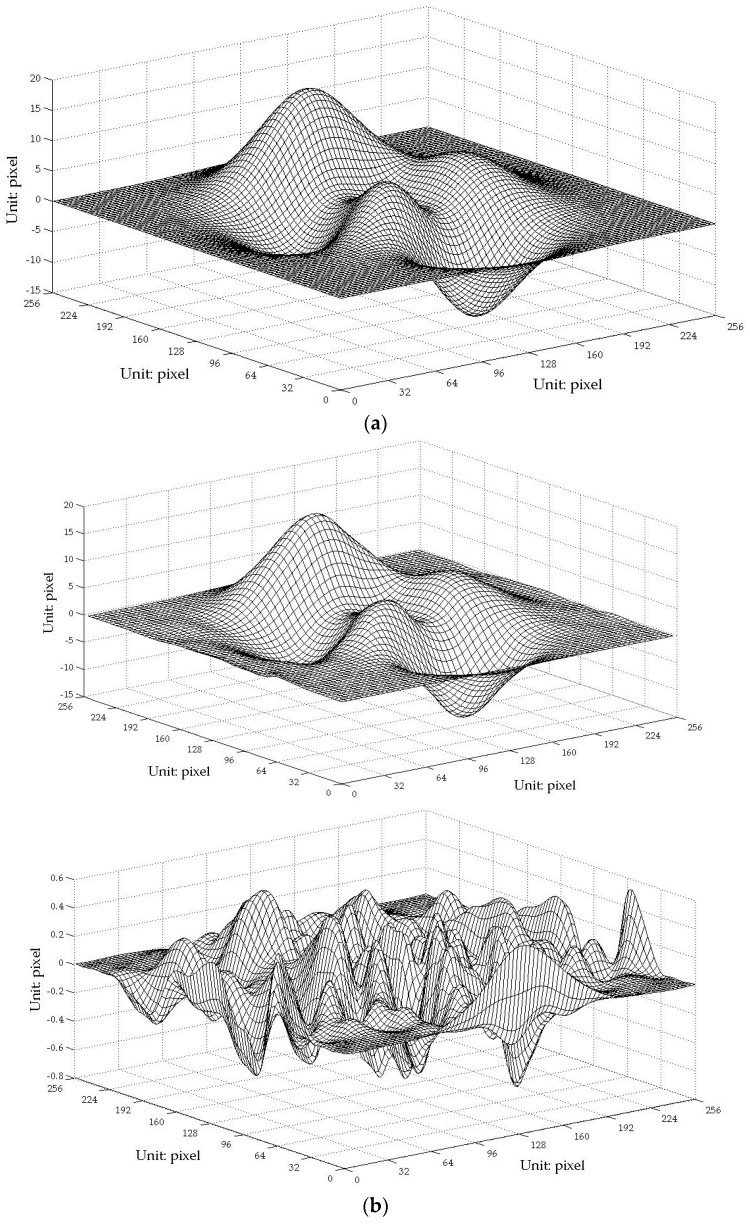

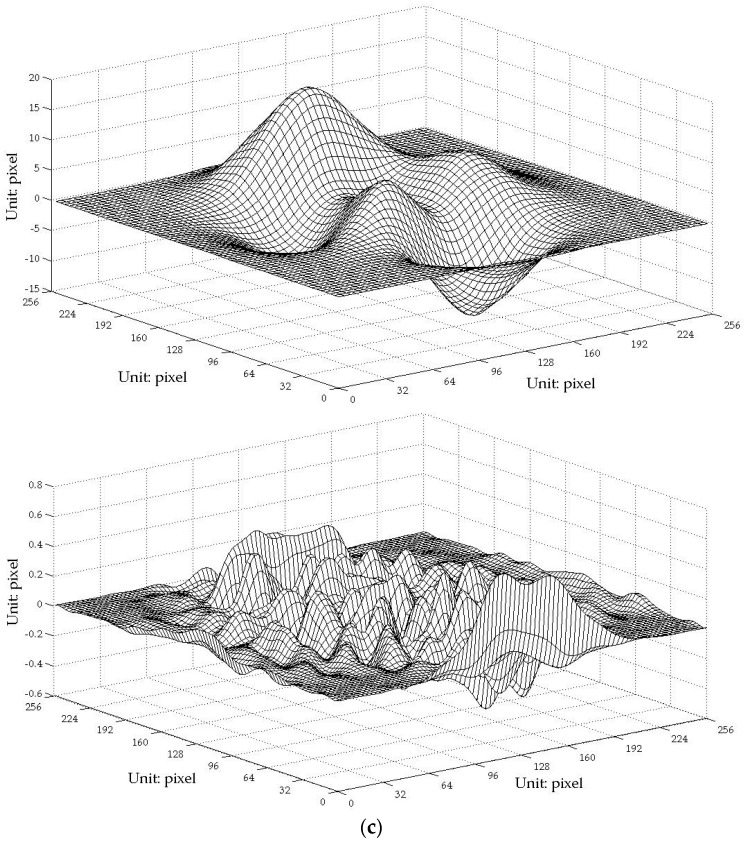
Simulation using the Peaks function. (**a**) The original image; (**b**) the up image shows the rebuilt profile using PMP method and the down image shows the errors between the 3D reconstruction and the original; (**c**) the up image shows the rebuilt profile using the one-shot CP technique and the down image shows the errors between the 3D reconstruction and the original.

**Figure 10:**
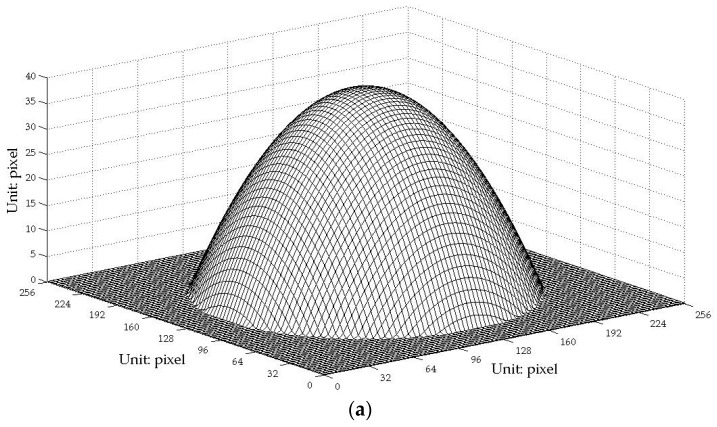

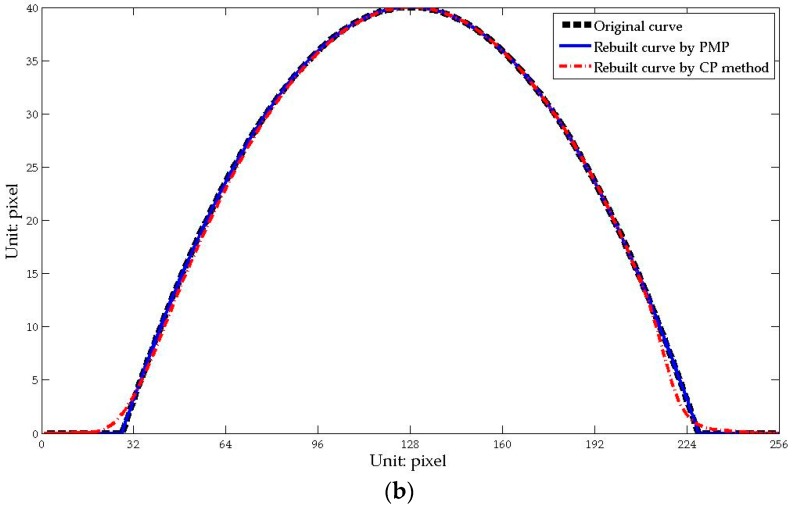
Simulation using an artificial upside-down bowl. (**a**) The original profile; (**b**) contour curves comparison of the two reconstructions against the original.

Furthermore, to find the errors between the 3D reconstruction and the original and to make a quantitative comparison between the traditional PMP method and the one-shot CP technique proposed in this paper, simulations were explored because the real digital information of the face model is hard to attain. The Peaks function included in the commercial MATLAB software as shown in Figure 9a and an artificial upside-down bowl as shown in Figure 10a were used for the studies. The results show that the rebuilt profiles have small errors against the original and no distinct decrease in the precision of the proposed one-shot CP method unlike the traditional phase-shifting method. However, we found that, around the edge of the bowl, there is a slightly greater deviation.

### 3.2. Survey of a Real Human Face

**Figure 11:**
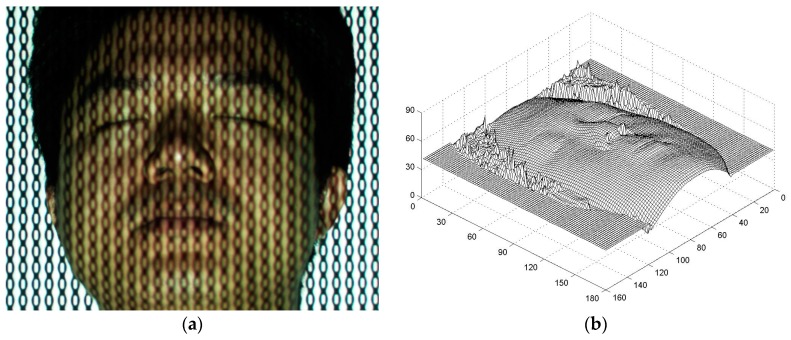
(**a**) Distortion composite pattern modulated by a volunteer’s face depth; (**b**) rebuilt 3D digital profile of a real human face (Scale: mm).

**Figure 12:**
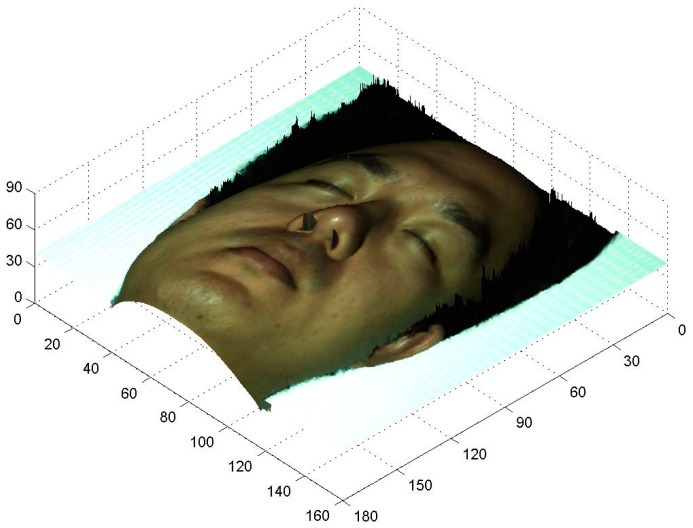
3D curved surface rebuilt based on the texture mapping technique (Scale: mm).

The aforementioned experimental setup was employed to survey a real 3D human face profile. The fundamental parameter $f_{\phi }$ was 1/25 line/pixel, whereas the other parameters remain the same. Figure 11a shows the distortion composite pattern modulated by the volunteer’s face depth, and Figure 11b is the 3D digital face profile rebuilt in the survey. The 3D digital data of a real human face were successfully obtained; although some noise was observed around the edge, which is acceptable. The texture mapping technique was applied to extract the color map of the real face and fuse it into the artificial face to reconstruct a nature-appearance 3D digital face [33,34,35,36]. Figure 12 shows the 3D curved surface rebuilt by using the texture mapping technique. The proposed technique enables a surgeon to extract 3D features and design an operation more easily and conveniently from the true face model.

## 4. Discussion

This study employs a composite pattern to obtain a 3D human face digital profile. This one-shot technique can avoid unwanted difficulties, such as trailing and distortion, which can occur in PMP when only one projection and a corresponding capture are required. A 3D digital model of a real human face can be obtained more conveniently and precisely based on the proposed approach. The rebuilt 3D profile of a real human face obtained in this study shows a suitable shape, except for some noise around the edge, which is acceptable. The texture mapping technique was also employed to reconstruct a nature-appearance 3D digital face. A true face 3D profile can provide more convenience to surgeons who must extract 3D face features and design a proper surgical operation.

The active optical 3D sensing techniques, such as the traditional PMP method and the one-shot CP method proposed in this paper, obtain the 3D information by unwrapping the phase from the observation light. As we know, the propagation of the wave can be regarded as the phase travels so that the distance of the wave moves (i.e. the wave-path) determines the phase value. While a sinusoidal fringe or a certain pattern that has a regularly distributed phase is projected to an uneven surface, the phase distribution in the reflected light is determined by the wave-path which is related to the height of the surface. However, two troubles to note are as follows: (1) the steep surface with discontinuities in surface slopes due to sharp edges will give rise to significant changes to the phase. In general, unwrapping the phase can be challenging, so added errors will be introduced to the results if the phase variations between the two adjacent sampling signals exceed one period, i.e., 2π. That is why the algorithm has a hard time around the edge of the specimen. (2) The phase information contained in the observation light would be disturbed if the reflector has very high or very low reflectivity, as the rippling pattern of brightening and dimming represents phase variety. A piece of white or low-contrast observation light caused by the high reflectivity adds difficulties to the phase unwrapping, so the rebuilt image would be in low grade.

In addition, the visible light projected from DLP generally makes a volunteer feel uncomfortable, especially on the eyes. Although the one-shot approach has cut the operation time, the eyes can be affected if they remain open during the survey. Thus, we asked the volunteer to close his eyes. The rebuilt shape with closed eyes can cause difficulties to the surgeon.

Our research group will then employ near-infrared (NIR) light generated by an NIR-DLP to project the composite pattern. An NIR-CCD will then be employed to capture the distortion pattern. People might be more comfortable in the NIR environment. Given this technique, a device called a Digital Face Beautifier can be developed to help surgeons produce tentative designs and inspect the effects before a surgical operation. This approach is more convenient to both the surgeon and the patient.

## 5. Conclusions

This paper reports on a novel active optical technique for 3D human face profile acquisition by utilizing a projection pattern composed of several frames of phase-shifting patterns and the same number of carrier patterns. Several techniques in the traditional method, such as phase-shifting or image transform, can also be utilized in this one-shot approach to solve the phase demodulation with automatic processing. The texture mapping technique can be applied to obtain a true 3D face profile. The face model test and real human face measuring prove that this method is simple, fast, and accurate. Therefore, a surgeon can utilize this approach to extract 3D appearance characteristics of a human face and to design operations more easily.
